# Endogenous trans-translation structure visualizes the decoding of the first tmRNA alanine codon

**DOI:** 10.3389/fmicb.2024.1369760

**Published:** 2024-03-04

**Authors:** David Teran, Ying Zhang, Andrei A. Korostelev

**Affiliations:** RNA Therapeutics Institute, UMass Chan Medical School, Worcester, MA, United States

**Keywords:** endogenous tmRNA, tmRNA decoding, A-minor interactions, SmpB, cryo-EM, alanyl-tRNA

## Abstract

Ribosomes stall on truncated or otherwise damaged mRNAs. Bacteria rely on ribosome rescue mechanisms to replenish the pool of ribosomes available for translation. Trans-translation, the main ribosome-rescue pathway, uses a circular hybrid transfer-messenger RNA (tmRNA) to restart translation and label the resulting peptide for degradation. Previous studies have visualized how tmRNA and its helper protein SmpB interact with the stalled ribosome to establish a new open reading frame. As tmRNA presents the first alanine codon via a non-canonical mRNA path in the ribosome, the incoming alanyl-tRNA must rearrange the tmRNA molecule to read the codon. Here, we describe cryo-EM analyses of an endogenous *Escherichia coli* ribosome-tmRNA complex with tRNA^Ala^ accommodated in the A site. The flexible adenosine-rich tmRNA linker, which connects the mRNA-like domain with the codon, is stabilized by the minor groove of the canonically positioned anticodon stem of tRNA^Ala^. This ribosome complex can also accommodate a tRNA near the E (exit) site, bringing insights into the translocation and dissociation of the tRNA that decoded the defective mRNA prior to tmRNA binding. Together, these structures uncover a key step of ribosome rescue, in which the ribosome starts translating the tmRNA reading frame.

## Introduction

Translation of messenger RNAs (mRNAs) into functional proteins is crucial for all living organisms. While the core translation mechanism is conserved across life kingdoms (Melnikov et al., 2012; Voorhees and Ramakrishnan, 2013; Korostelev, 2022), adaptation to different conditions has led to the evolution of distinct translational control strategies (Buskirk and Green, 2017). One challenge encountered by translating ribosomes is the truncation or other damage of mRNA molecules, resulting in ribosome stalling at the truncation or damage site. In growing *E. coli*, between 0.4% (Moore and Sauer, 2005) and 4% (Ito et al., 2011) of mRNAs are estimated to be damaged at a given time. Several strategies have evolved to “rescue” stalled ribosomes and replenish the pool of active ribosomes (Keiler, 2015; Korostelev, 2021; Muller et al., 2021; Kurita and Himeno, 2022). Trans-translation, the main strategy conserved among eubacteria, allows the ribosome to switch from the damaged mRNA to a different open reading frame, targeting the mRNA and incomplete peptide for degradation and completing translation on a conventional stop codon (Komine et al., 1994; Tu et al., 1995; Keiler et al., 1996; Karzai et al., 1999; Yamamoto et al., 2003). Perturbation of trans-translation in most eubacteria leads to the accumulation of stalled ribosome complexes and inability to recover from stress (Janssen and Hayes, 2012; Schopping et al., 2022).

**Figure 1:**
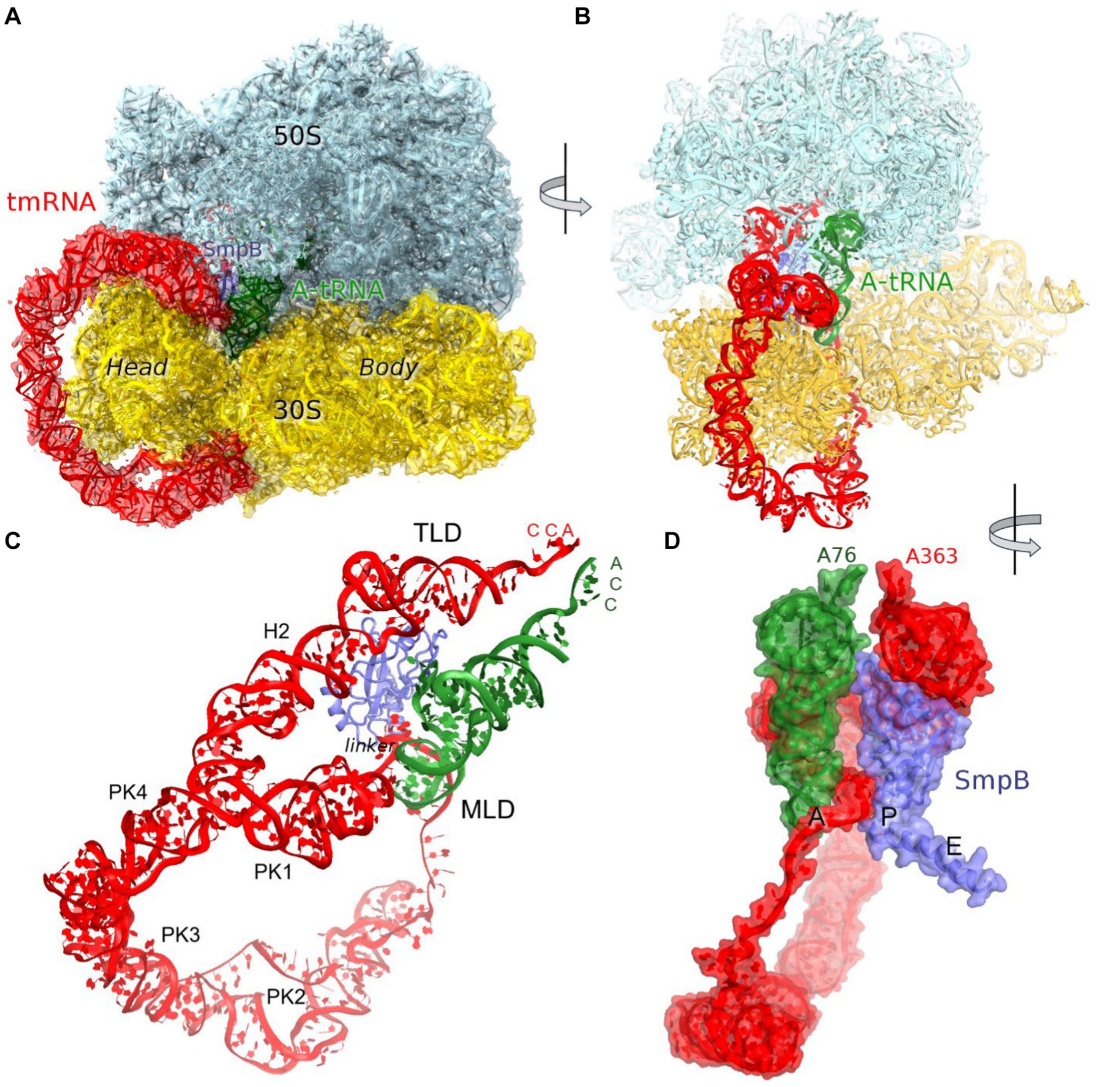
Cryo-EM structure of *Escherichia coli* 70S•tmRNA complex with tRNA^Ala^ in the A site. **(A)** 3.7-Å cryo-EM density segmented to show the ribosomal subunits (cyan and yellow), tmRNA (red), SmpB (purple) and A-site tRNA (green). **(B)** Front view of the 70S structure with tmRNA, SmpB and A-tRNA; **(C)** Relative positions of tmRNA, SmpB and A-tRNA, with tmRNA domains labeled. **(D)** Close-up view of tmRNA, SmpB and A-tRNA facing the CCA ends of the tRNA and TLD (in the A and P sites, respectively) and SmpB C-terminus in the E site, rendered as molecular surfaces.

Trans-translation is accomplished by a hybrid transfer-messenger RNA (tmRNA), comprising a tRNA-like domain (TLD), an mRNA-like domain (MLD), and four pseudoknots (PK1 through PK4) that form a circularized structure (Figures 1A,B; Janssen and Hayes, 2012; Giudice and Gillet, 2013). The TLD, comprising a tRNA-like acceptor arm charged with alanine, associates with small protein B (SmpB), which functionally mimics the tRNA’s anticodon stem loop (Komine et al., 1994; Ushida et al., 1994; Karzai et al., 1999; Gutmann et al., 2003; Weis et al., 2010). The MLD contains a short internal open reading frame, which connects with the TLD via PK1 and a single-stranded linker (Figure 1C).

Trans-translation starts with the binding of the TLD•SmpB complex to the ribosomal A site followed by EF-G-catalyzed translocation of TLD•SmpB to the P site. These steps have been characterized by X-ray crystallography and cryo-EM of *in vitro* assembled tmRNA-bound ribosomes, bringing key insights into the recognition of the stalled ribosomes, formation of the TLD-peptide complex, and tmRNA rearrangements upon translocation (Weis et al., 2010; Neubauer et al., 2012; Ramrath et al., 2012; Rae et al., 2019; Guyomar et al., 2021). The alanyl-TLD is delivered to the ribosome by EF-Tu, similarly to canonical amino-acylated tRNAs (Neubauer et al., 2012; Miller and Buskirk, 2014). Upon accommodation of the TLD in the A site of the 50S subunit, the stalled peptide is transferred to the alanine residue on tmRNA. The C-terminal helix of SmpB initially binds in the vacant mRNA entry tunnel of the 30S subunit to recognize the ribosomes with truncated mRNAs. During translocation, SmpB moves along with the TLD to the P site, while its C-terminal helix “leaps” into the E site, thus freeing the A site (Rae et al., 2019; Guyomar et al., 2021). This allows the MLD of tmRNA to present the first codon—GCA coding for alanine—for recognition by the canonical alanyl-tRNA^Ala^. Cryo-EM studies demonstrated tRNA binding to the A site in the presence of tmRNA in *E. coli* and *M. smegmatis* (Fu et al., 2010; Mishra et al., 2018), however low > 12 Å resolutions prevented detailed characterization of this trans-translation step. Recent higher-resolution cryo-EM structures of the translocated tmRNA with a vacant A site showed that tmRNA linker traverses the A site, partially blocking the canonical tRNA binding location (Rae et al., 2019; Guyomar et al., 2021). The tmRNA therefore must reorganize to allow the binding of tRNA^Ala^ and translation of the tmRNA coding sequence.

In this work, we describe a tmRNA-bound complex that copurified with *E. coli* 70S ribosomes and features an endogenous tmRNA structure (Methods). The complex contains tRNA^Ala^ in the A site, stabilized by interactions with tmRNA and SmpB. A fraction of this complex also contains a deacyl tRNA near the E site, revealing a non-canonical tRNA binding site that may be sampled in a preceding step of tmRNA translocation.

## Results and discussion

### Cryo-EM structure of the 70S•tmRNA•SmpB complex with A-site tRNA

We performed maximum-likelihood classification of a large cryo-EM dataset (~1.5 million particles) collected from 70S ribosomes that were purified from *E coli* at the exponential growth phase and then incubated with defined mRNA, tRNA^Phe^, tRNA^fMet^ and stringent factor RelA (Supplementary Figure S1; Methods). Remarkably, we found that ~14,000 of the 1.15 million 70S ribosome particles contain tmRNA (~1.2%). Because neither tmRNA nor tRNA^Ala^ were added to the 70S sample, the tmRNA-bound ribosomes must represent endogenous *E. coli* trans-translation complexes. Since the tmRNA-bound ribosomes formed prior to the addition of RelA (Methods), they likely represent a homeostatic trans-translation complex. Indeed, the 1.2% recovery of trans-translation ribosomes comports with the cellular estimates of rescue-complex abundance (Moore and Sauer, 2005; Ito et al., 2011).

**Figure 2:**
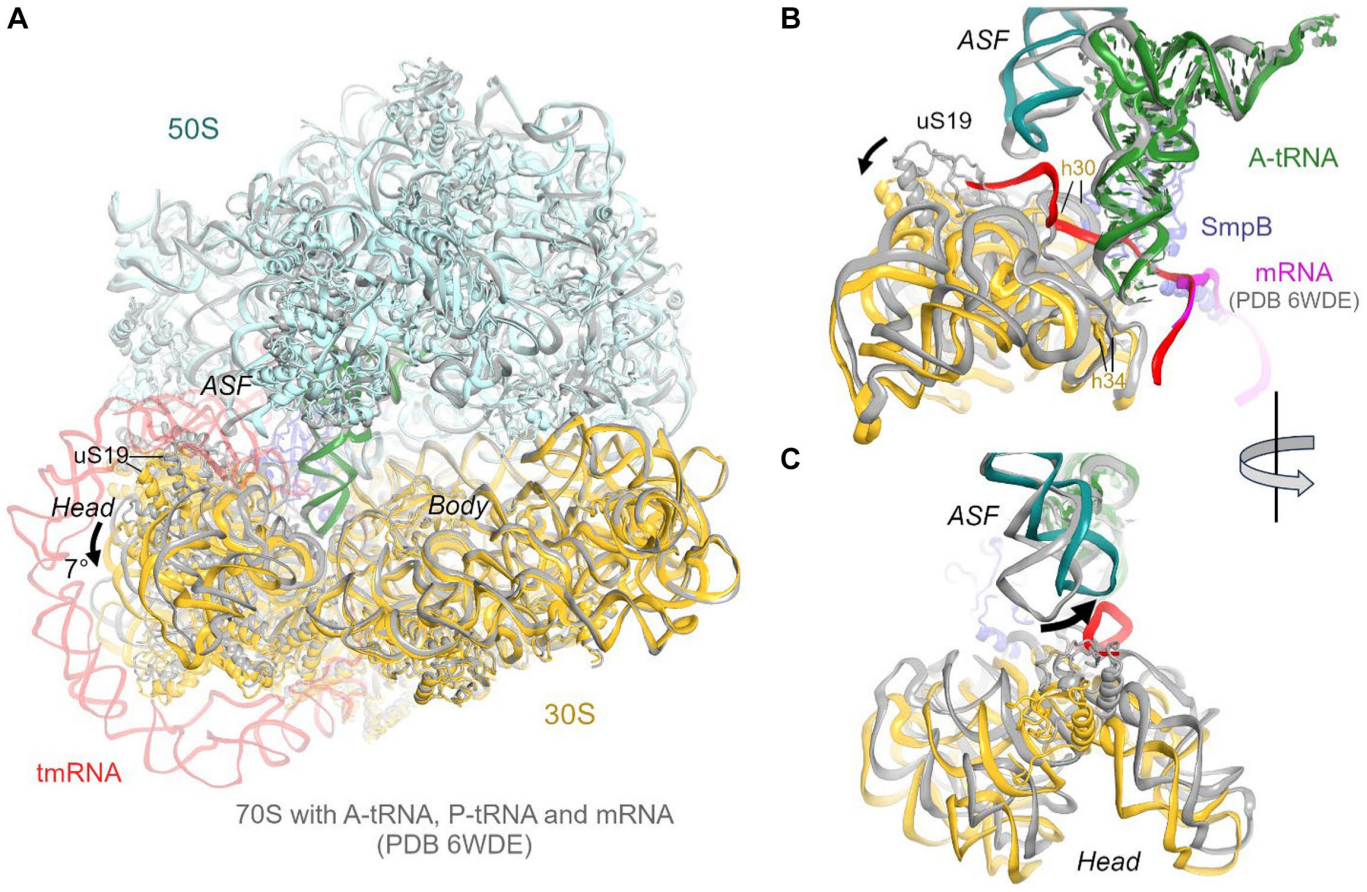
Comparison of the 70S•tmRNA•tRNA^Ala^ complex with the canonical *Escherichia coli* 70S elongation complex bound with mRNA and three tRNAs (PDB 6WDE). **(A)** Superposition of the 70S•tmRNA•tRNA^Ala^ structure (colored) with the 70S•mRNA•tRNA_3_ structure (gray) shows overall similar ribosome conformations except for the 7° tilt of the 30S head domain. Structures were superposed by aligning 23S rRNA (here and in other figures). **(B)** Close-up view of the similarly positioned tRNA in the A site of the tmRNA-bound (colored) and canonical elongation-state (gray) ribosomes. **(C)** Close-up view showing different positions of the A-site finger (ASF; H38 of 23S rRNA) in the tmRNA-bound (colored) and canonical elongation-state (gray) ribosomes.

The predominant 3.7 Å cryo-EM reconstruction with circularized tmRNA density features a non-rotated ribosome with strong densities for TLD•SmpB in the P site and tRNA in the A site (Figure 1A; Supplementary Figure S1). Our extensive classification did not identify tmRNA in other ribosome sites, similarly to the recent study of *M. smegmatis* ribosomes (Mishra et al., 2018), suggesting that this complex represents an intermediate accumulating during trans-translation. The ribosome and the endogenous tmRNA, wrapped around the head of the 30S subunit (Figure 1B), are overall similar to those in post-translocation 70S complexes assembled from *in vitro* transcribed tmRNA constructs (Rae et al., 2019; Guyomar et al., 2021). The ribosomal intersubunit rotation state during elongation correlates with stages of decoding and translocation (Cornish et al., 2008; Ermolenko and Noller, 2011; Rodnina, 2018). The non-rotated ribosome with A-site tRNA corresponds to a post-decoding stage, preceding the translocation of the tRNA into the P site that requires intersubunit rotation. In addition, translocation involves a “swiveling” motion of the head domain of the 30S subunit (Ratje et al., 2010). Another mode of the head movement, known as “tilt,” normally occurs during initiation, when the free 30S subunit interacts with the initiator tRNA sampling the P site (Hussain et al., 2016; Jahagirdar et al., 2020). While mechanistically similar to the elongation ribosomes with P- and A-site tRNAs, the tmRNA-bound ribosome is reorganized via a head tilt to accommodate the bulky tmRNA. As tmRNA helix 2 and pseudoknot 1 are placed between the 30S head and the 50S central protuberance (Figures 1B,C), the head is tilted 7° away from the large subunit placing uS19 ~ 15 Å farther than in canonical elongation complexes (measured at Gly25; Figures 2A,B). The A-site finger (ASF) of the large subunit, involved in tRNA accommodation (Sanbonmatsu et al., 2005; Loveland et al., 2020), is shifted by ~20 Å (measured at the U887 tip) to dock onto PK1 (Figure 2C).

The structure brings insight into tRNA positioning in the A site of the tmRNA-bound ribosome. On the 50S subunit, despite the large shift of the 50S ASF, the A-site tRNA elbow is stabilized by packing against the ASF (Figure 2B). Here, the C19-G56 pair likely stacks on the bulged A896 of the ASF (Supplementary Figure S2A), similarly to canonical tRNA-bound complexes. Accordingly, the position of the A-site tRNA relative to the 50S subunit is nearly identical to those in tRNA-bound structures, emphasizing the invariant mechanism of tRNA accommodation for peptidyl transfer. The acceptor arm with the 3′ terminal CCA is inserted into the A site next to the CCA end of the tmRNA (Figure 1D). In the polypeptide tunnel, scattered density suggests compositional and conformational heterogeneity of peptides in the endogenous rescue complexes on different mRNAs.

### Decoding of the tmRNA alanine codon

Due to the 30S head tilt, interactions between the A-site tRNA and the 30S subunit slightly differ from those in canonical tRNA-bound complexes. Whereas helix 30 of 16S rRNA normally binds near the anticodon stem of tRNA (at nt 42), helix 30 is retracted by ~9 Å (measured at U956) to accommodate the tmRNA linker connecting PK1 and MLD. Universally conserved C1054, which bulges from h34, normally buttresses the anticodon by packing on the ribose of nt 34. But in the tmRNA-bound complex, the 30S head tilt shifts C1054 by ~4 Å, detaching it from tRNA^Ala^ (Figure 2B).

**Figure 3:**
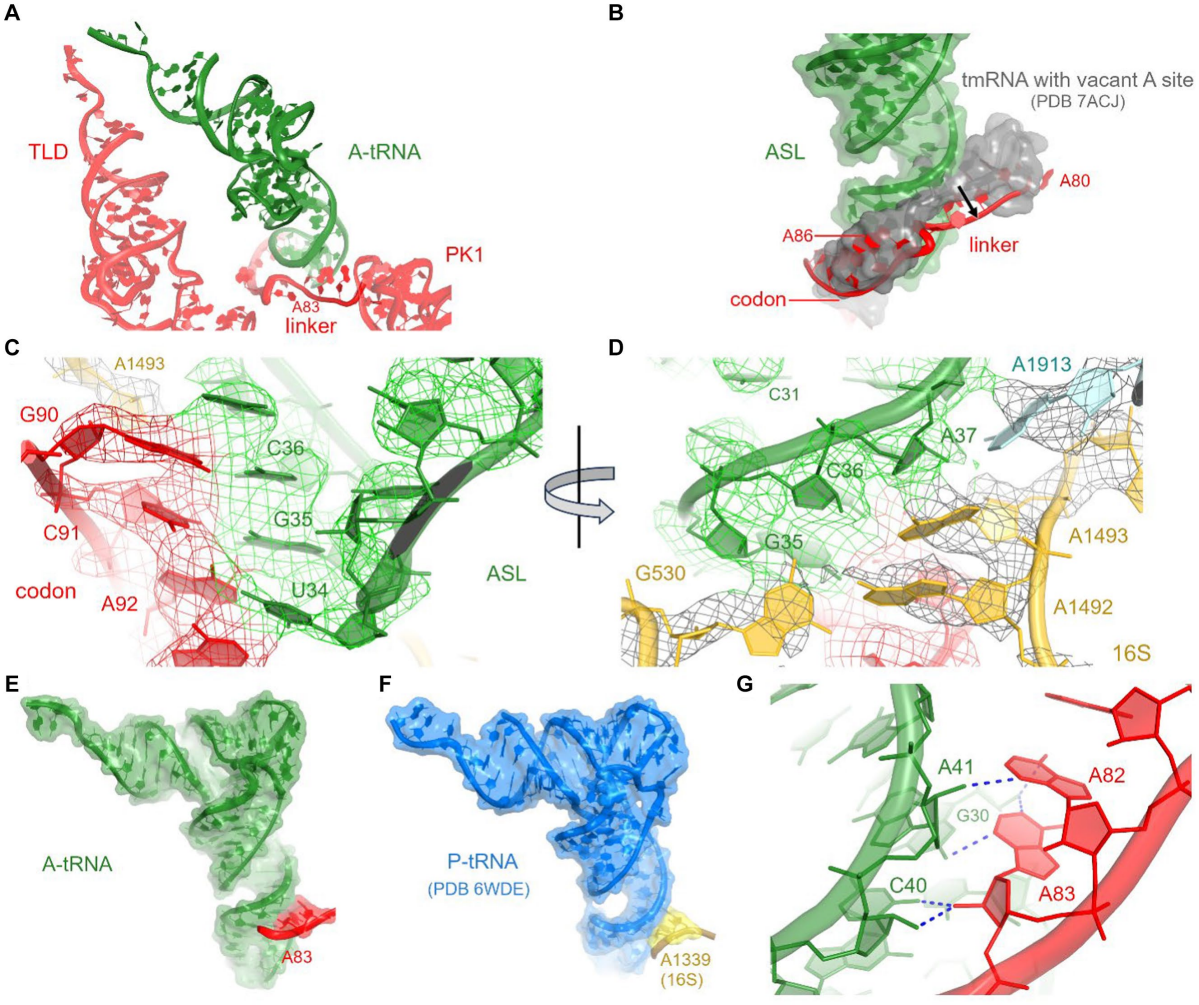
Interactions between A-tRNA and tmRNA. **(A)** Positions of A-tRNA and tmRNA domains. **(B)** The tmRNA linker (red) shifts to accommodate the A-site tRNA, relative to its position in the tmRNA-bound complex with a vacant A site (gray backbone and molecular surface) in PDB 7ACJ. **(C,D)** Cryo-EM density in the decoding center, showing codon-anticodon interactions **(C)** and interactions with 30S and 50S nucleotides **(D)**. **(E)** Interactions of the tmRNA linker with the anticodon stem of A-tRNA resemble A-minor interactions (E) and are similar to those between 16S rRNA and tRNA^fMet^ in the P site **(F)** of initiation and elongation ribosome structures (PDB 6WDE is shown). **(G)** Close-up view of the A-minor-like interactions between tmRNA nucleotides and A-site tRNA.

The loss of interactions between tRNA^Ala^ and the 30S head is partially compensated by interactions with tmRNA and SmpB (see below), firmly positioning tRNA^Ala^ in the 30S decoding center. Local density confirms tRNA^Ala^-specific nucleotides and Watson-Crick base pairing of the tRNA UGC anticodon with the corresponding GCA codon of tmRNA (Figure 3C; Supplementary Figures S2B,C). The codon-anticodon helix is stabilized by interactions with ribosomal decoding-center nucleotides G530, A1492, and A1493 (Figure 3D). The G530 loop of the shoulder domain is disengaged from the h34 of the shifted head, unlike in canonical tRNA-bound ribosomes where A532 packs on G1207. Despite this difference, G530 stabilizes the tRNA anticodon by hydrogen bonding with the ribose of G35, and both nucleotides are placed nearly identical to those in canonical tRNA-ribosome complexes. The adenosines A1492 and A1493 of the body domain stabilize the opposite side of the codon-anticodon helix by hydrogen-bonding with the riboses of tmRNA codon nucleotides G90 and C91. Thus, the ribosome recognizes and stabilizes the tmRNA-tRNA^Ala^ codon-anticodon helix via the universally conserved G530 and A-minor interactions, as in canonical elongation complexes (Ogle et al., 2001; Demeshkina et al., 2012; Loveland et al., 2017).

To accommodate tRNA^Ala^ in the A site, the adenosine-rich linker of tmRNA (^80^AAAAAU^85^) shifts away from its position in pre-decoding structures (Rae et al., 2019; Guyomar et al., 2021), where the linker traverses the A site (Figures 3A,B). Adenosines A82 and A83 support the minor groove of the tRNA^Ala^ anticodon stem at nucleotides C40 and A41 (Figures 3E,G). This interaction appears similar to the A-minor-like interaction in the 30S P site, where conserved 16S nucleotides G1338 and A1339 pack at the minor groove of initiator tRNA^fMet^ (Figure 3F) to assist translation initiation and perhaps other translation stages (Lancaster and Noller, 2005; Korostelev et al., 2006; Hussain et al., 2016). A-minor-like interactions are a unique tertiary structure that plays critical roles in RNA stabilization, including tetraloop-receptor recognition (Cate et al., 1996; Doherty et al., 2001; Nissen et al., 2001; Battle and Doudna, 2002) and mRNA decoding described above. Furthermore, their modest interaction surface and thermodynamic stability (Doherty et al., 2001) allow for local structural rearrangements, such as tRNA dynamics during mRNA decoding and translocation (Ogle et al., 2001; Demeshkina et al., 2012; Loveland et al., 2017; Carbone et al., 2021). Thus, the tmRNA linker not only replaces the tRNA interactions with the 30S head during decoding but may also transiently stabilize tRNA^Ala^ and/or disengage from the tRNA^Ala^ in the subsequent—highly dynamic—translocation step. The functional role of this A-minor interaction is supported by the conservation of adenosines positioned 6–10 nucleotides upstream of the first codon of tmRNA in most bacterial species (Zwieb et al., 1999). In species without consecutive adenosines in this position (e.g., *Thermus thermophilus* tmRNA), however, it remains to be seen how tmRNA interacts with tRNA^Ala^ (Op De Bekke et al., 1998; Kaur et al., 2006).

SmpB adopts the same overall conformation as in the structures without A-site tRNA (Rae et al., 2019; Guyomar et al., 2021). Here, the protein’s globular domain binds the 30S P site, with the His79 loop sandwiched between TLD and the elbow of tRNA^Ala^. To accommodate the tRNA, the loop is slightly rearranged, bringing His79 into contact with the tRNA^Ala^ backbone at nucleotide 17. The C-terminal helix occupies the mRNA binding pocket in the E site (Figure 1D).

### tRNA in the remodeled E site

**Figure 4:**
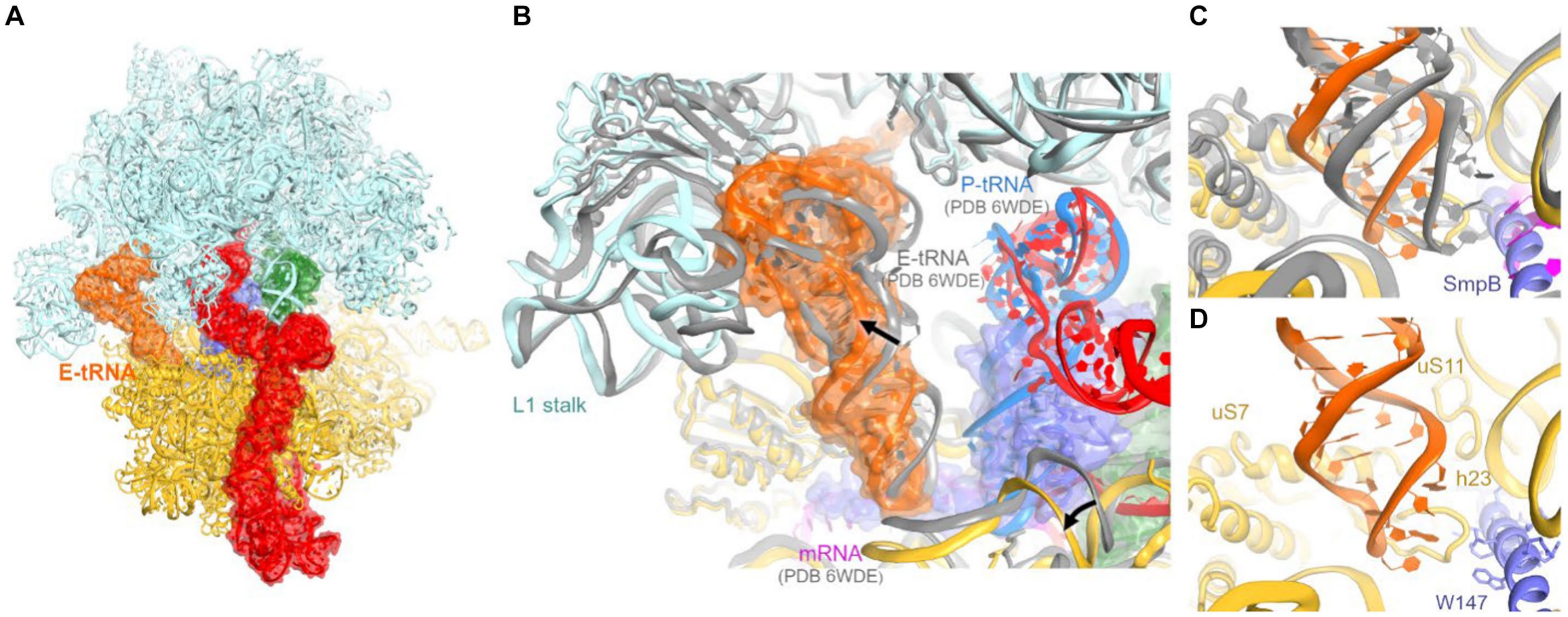
Cryo-EM structure of *E. coli* 70S•tmRNA complex with tRNAs in the A (green) and E (orange) sites. **(A)** Front view of the 70S structure with tmRNA, SmpB, A-tRNA and E-tRNA rendered as molecular surfaces. **(B)** Position of the tRNA in the E site of the 70S•tmRNA•tRNA^Ala^ complex (colored) differs from that in the canonical 70S•mRNA•tRNA_3_ structure (gray ribosome and E-tRNA, magenta mRNA and blue P-tRNA). **(C,D)** Positions of the anticodon stem loops of E-site tRNAs relative to 16S and SmpB (in the 70S•tmRNA•tRNA^Ala^ complex) or mRNA (in the 70S•mRNA•tRNA_3_ complex).

In our tmRNA-containing cryo-EM reconstructions, low-density features in the E site suggested sub-stoichiometric tRNA. To better resolve this density, we subclassified the cryo-EM maps, using a mask covering the E site, yielding a 3.9-Å resolution class with tRNA near the canonical E site (Figure 4A; Supplementary Figures S1, S2D). Other complex constituents, including the A-site tRNA, are similar to the complex described above.

Interactions of the tRNA with the 30S E site differ from canonical ribosome structures with an untilted head domain. In canonical complexes, E-site tRNA binds in the cleft between the head (near ribosomal protein uS7) and body (near G693 of h23 of 16S rRNA). In the tmRNA-containing complexes, however, the tRNA is shifted ~8 Å away from this binding pocket, despite ample space near the C-terminal helix of SmpB (Figures 4B–D). Furthermore, the tRNA is partially retracted from the E site of the head, formed by h29 of 16S rRNA and uS7. Here, the anticodon is shifted ~9 Å away from the tip of the β-hairpin of uS7 (at Gly80), where the tRNA anticodon resides in elongation complexes (Figures 4C,D). In this position, the anticodon stem loop is loosely held near h29 (at A1339) and the β-sheet (at Arg78) and C-terminal α-helix (near Arg142) of uS7. On the 50S subunit, the tRNA interacts with the L1 stalk (elbow) and H88 of 23S rRNA, where the terminal nucleotide A76 is inserted into. These contacts are nearly identical to those observed in numerous structural studies of tRNA-bound complexes (Figure 4B).

Previous structural studies of the P-site bound tmRNA did not report E-site tRNA, suggesting that tRNA bound to the preceding truncated mRNA readily dissociates upon tmRNA translocation. The tRNA in our map likely results from the addition of tRNA^Phe^ and tRNA^fMet^ to the ribosome sample (Methods). Nevertheless, this observation may report a transient tRNA binding state sampled during or immediately after tmRNA translocation. The structure underlines that unlike the A and P site, where the tRNA interacts closely with both the 30S and 50S subunits, E-site positioning is driven primarily by interactions with the 50S subunit. Indeed, these interactions are established during the initial stages of translocation, in which the acceptor arm of deacyl-tRNA is spontaneously transferred from the P site to the E site on the 50S subunit, while the anticodon stem loop remains bound to the 30S subunit (Agirrezabala et al., 2008; Fei et al., 2008; Julian et al., 2008; Cornish et al., 2009). In addition, non-canonical E-like tRNAs were found in other studies of bacterial ribosomes (E-out; Zhang et al., 2018) and eukaryotic ribosomes (Z-site; Brown et al., 2018). While retaining the invariant interactions with the large-subunit L1 stalk and the CCA-binging pocket, these structures feature different tRNA interactions with the small subunit. Distinct binding modes allow for increased tRNA dynamics in the E site, underlying the tRNA-dissociation function of the E site (Brown et al., 2018; Zhang et al., 2018). Our findings expand the repertoire of possible tRNA rearrangements during tRNA departure from the ribosome.

In conclusion, our cryo-EM analyses visualize how the first tmRNA codon is decoded and how tmRNA rearranges to accommodate tRNA^Ala^. Interactions with tmRNA stabilize tRNA^Ala^, which binds nearly identically to canonical A-site tRNA despite a substantial tilt of the 30S head. Future work will detail whether A-minor-like interactions of tmRNA with the tRNA anticodon stem occur in bacterial species, whose tmRNA linker does not contain continuous adenosines. Further, rearrangements of these and other interactions of tmRNA•SmpB with tRNA^Ala^ and the ribosome during translocation of tRNA^Ala^ to the P site remain to be visualized. Such structural studies may inform the development of new drugs that target trans-translation, a promising target for antibacterial therapeutics (Campos-Silva et al., 2021; Marathe et al., 2023).

## Materials and methods

### 70S ribosome preparation

70S ribosomes were prepared from MRE600 *E. coli* essentially as described (Moazed and Noller, 1986, 1989) and stored in the ribosome-storage buffer A (20 mM HEPES (pH 7.5), 100 mM KCl, 10.5 mM MgCl_2_, 0.5 mM EDTA, 5 mM β-mercaptoethanol) at −80°C. In short, MRE600 *E. coli* stock was grown on LB agar plates at 37°C. Then, one colony from the plate was inoculated in 100 mL LB media and grown at 37°C overnight in an incubated shaker at ~220 rpm. Forty eight milliliters of overnight culture was inoculated into 6 L LB media and the culture was incubated at 37°C to mid-log phase (OD_600_ 0.5–1.0).

*Escherichia coli* cells obtained from a 6 L culture were suspended in 50 mL cold buffer A and lysed using a microfluidizer (Microfluidics, United States) at 18 k psi. The lysate was clarified using a JA-20 rotor at 39,200 × *g*, 4°C, for 20 min. The clarified supernatant was layered onto 35 mL (per tube) of 37.7% sucrose in buffer A. Ribosomes were sedimented onto the sucrose cushion by ultracentrifugation in a 45 Ti rotor at 185,677 × *g* (40,000 rpm), 4°C, for 20 h. The ribosome pellet was dissolved in 2 mL of buffer A. The ribosome solution was transferred to 1 mL microcentrifuge tubes and spun at 13 K rpm at 4°C for 10 min. Supernatant was transferred to a 50 mL tube, the volume was adjusted to 40 mL using cold buffer B (70 mM Tris–HCl (pH 7.0), 500 mM NH_4_Cl, 15 mM MgCl_2_, 0.5 mM EDTA, 5 mM β-mercaptoethanol), and ribosomes were sedimented in 70 Ti tubes at 310,801 × *g* (55,000 rpm), 4°C, for 2 h. The ribosome pellet was resuspended in 1 mL buffer A, aliquoted and stored at −80°C.

The ribosome complex for cryo-EM analyses was prepared as described (Loveland et al., 2016) with some modifications. 70S ribosomes at a final concentration of 0.4 μM were activated in 20 mM HEPES-KOH (pH 7.5), 120 mM KCl, 15 mM MgCl_2_, 2 mM spermidine, and 0.05 mM spermine at 42°C for 15 min. Activated ribosomes were incubated with 0.8 μM mRNA (5′ GGCAAGGAGGUAAAAAUGUUCAAAAAA 3′), 0.8 μM tRNA^fMet^, and 1 μM tRNA^Phe^ (all final concentrations) at 37°C for 30 min. The sample was then incubated with 4 μM RelA, 15 μM Adenosine-5′-[(α,β)-methyleno] triphosphate APCPP; Thermo scientific and 15 μM Guanosine triphosphate (GTP; Thermo scientific) to assemble a stringent-response 70S complex, for 15 min at room temperature. The final volume of the sample was 30 μL.

### Cryo-EM grid preparation and data collection

Carbon-coated EM grids (Ultrathin Carbon on Quantifoil®, 2 μm Diameter Holes, 1 μm Separation, mounted on a 200 M Cu grid coated with a 2-nm thin layer of carbon; TedPella) were glow discharged at 20 mA with a negative polarity setting for 30 s in a PELCO easiGlow glow discharge unit. 3 μL of the 70S sample was applied to the grid. Grids were blotted for 4 s with a blotting force of 7 and plunged into liquid ethane using a Vitrobot Mark IV (ThermoFisher Scientific), whose chamber was pre-equilibrated to 4°C and 95% humidity.

Two data sets were collected on a UMass Chan Cryo-EM Facility Talos Arctica electron microscope (ThermoFisher Scientific) operating at 200 kV and equipped with a K3 direct electron detector (Gatan Inc.) targeting 0.55–1.1-μm underfocus. Data were collected using SerialEM (Mastronarde, 2005), with beam tilts to record several movies at each stage position. The datasets contain 4,016 movies (total dose of 29.9 e^−^/Å^2^ on the sample), yielding 474,262 particles and 8,021 movies (30.4 e^−^/Å^2^ on the sample), yielding 1,039,487 particles. Movies were aligned during data collection using IMOD (Kremer et al., 1996) to decompress frames, apply the gain reference, and to correct for image drift and particle damage and bin the super-resolution pixel by 2.

### Cryo-EM data processing

CTF parameter determination, reference-free particle picking, and stack creation were carried out in cisTEM (v1.0-beta; Grant et al., 2018). Particle alignment and refinement were carried out in FREALIGNX (Lyumkis et al., 2013). Data processing was initially performed independently for each dataset (Supplementary Figure S1A). To speed up the processing, 2×− and 4×−image stacks were prepared using resample.exe, which is part of the FREALIGN distribution (Lyumkis et al., 2013). The initial model for particle alignment of 70S maps was the 11.5 Å resolution EMDB-1003 (Gabashvili et al., 2000), sampled to match 4×−binned image stack using resample.exe. Three rounds of mode 3 search alignment to 25 Å were run using the 4×−binned stack. Next, 25–30 rounds of mode 1 refinement were run with the 4×−binned, 2×−binned, and eventually unbinned stacks until the resolutions stopped improving, to the final resolutions of 2.8 Å and 2.7 Å of the overall maps. 3D maximum-likelihood classification into 20 classes was performed in FREALIGN v9.11 to separate 70S conformations, 50S subunits, and junk (poorly aligned or damaged) particles. An unexpected class emerged in each stack, featuring density near the 30S head, which connects the mRNA tunnel with the A-site finger and P site. The 70S classes with different tRNA occupancies and ribosome conformations (including the tmRNA class) were extracted into a stack per data set, using merge_classes.exe from FREALIGN distribution. Two 70S stacks were merged using IMOD 4.7 (Kremer et al., 1996).

The merged 70S stack was refined as described above, yielding a final average 70S reconstruction at 2.8 Å resolution. The refined parameters were used to run a 3D maximum-likelihood classification into 32 classes without a mask, with an ASF-covering mask, or with the A-site-covering mask. All masks were “spherical,” also known as “2D” masks on micrographs (Grigorieff, 2016), as opposed to specifically shaped “3D” masks (Supplementary Figure S1A). The tmRNA class was found in the no-mask and ASF-mask classifications. Particles with tmRNA density resulting from the ASF-mask classification were extracted into a substack. The tmRNA substack was classified at 4× with a P- and A-site covering mask or the E-site mask to further purify the tmRNA-containing density (P-A mask) or the E-tRNA-containing density (E mask). The unbinned stack was used to yield the resulting cryo-EM reconstructions with tmRNA (with A-tRNA and partial E-tRNA) and with tmRNA (with full-occupancy A-tRNA and E-tRNA) at resolutions of 3.7 Å and 3.9 Å, respectively (Supplementary Figure S1A).

Fourier Shell Correlation (FSC) curves were calculated by FREALIGNX for even and odd particle half-sets (Supplementary Figure S1B). The maps used for structure refinements was B-factor sharpened using the B factor of −100 Å^2^ up to 3.4 Å (tmRNA with A-tRNA) and −50 Å^2^ to 3.8 Å (tmRNA with A-tRNA and E-tRNA), using bfactor.exe (included with the FREALIGN distribution; Lyumkis et al., 2013).

### Structural model building and refinement

Structure of the non-rotated 70S•tRNA_3_ complex (V-B; PDB 6WDE; Loveland et al., 2020) and structures of 70S•tmRNA complexes (PDB: 6Q98 and 7ACJ; Rae et al., 2019; D’Urso et al., 2023) were used as starting models for 70S ribosome and tmRNA fitting, respectively. The model of tRNA^Ala^ (GGC anticodon) from PDB:6OF6 (Nguyen et al., 2020) was modified to fit the cryo-EM map and match the nucleotide sequence of tRNA^Ala^ (UGC). The 50S, 30S and tmRNA domains were fitted using UCSF Chimera 1.6 (Goddard et al., 2018; Pettersen et al., 2021) and locally modeled in Pymol1.2r1 (DeLano, 2002). The fitted structures were refined conservatively, using secondary-structure restraints and low simulated-annealing temperatures (100 K, 300 K or 500 K), against cryo-EM maps using phenix.real_space_refine v1.19.2 (Adams et al., 2010). Refinement parameters, such as the relative weighting of stereochemical restraints and experimental energy term, were optimized to produce the optimal structure stereochemistry and real-space correlation coefficients (Supplementary Table S1). B-factors of the models were refined at the final stages using phenix.real_space_refine. Structure stereochemistry validation was performed using phenix.molprobity.

Structure superpositions and distance calculations were performed in PyMOL. To calculate the angles of the 30S rotation and head tilt, 23S rRNAs of corresponding structures were aligned using PyMOL, and the angle between 16S domains were measured in Chimera. Figures were prepared in PyMOL and Chimera.

## Data availability statement

The original contributions presented in the study are included in the article/Supplementary material, further inquiries can be directed to the corresponding author. The structural models generated in this study have been deposited in the RCSB Protein Data Bank under the following accession codes: 8VSA (https://www.rcsb.org/structure/8VSA; 70S with tmRNA, SmpB and A-site tRNA^Ala^) and 8VS9 (https://www.rcsb.org/structure/8VS9; 70S with tmRNA, SmpB, A-site tRNA^Ala^ and E-site tRNA). The cryo-EM maps described in this study have been deposited in the Electron Microscopy Database under the following accession codes: EMD-43491 and EMD-43490, for the complexes without and with E-site tRNA density, respectively.

## Author contributions

DT: Data curation, Formal analysis, Investigation, Methodology, Validation, Visualization, Writing – original draft, Writing – review & editing, Conceptualization. YZ: Methodology, Resources, Writing – review & editing. AK: Conceptualization, Formal analysis, Funding acquisition, Investigation, Methodology, Project administration, Resources, Supervision, Validation, Visualization, Writing – original draft, Writing – review & editing.
