# Supplementary material for: Endogenous trans-translation structure visualizes the decoding of the first tmRNA alanine codon
Source: Front Microbiol. 2024 Mar 4;15:1369760. doi: 10.3389/fmicb.2024.1369760 (PMC10944890; doi:10.3389/fmicb.2024.1369760)
Supplement: Supplementary file 1 [file Data_Sheet_1.docx]

**Supplementary Materials for:**

Endogenous trans-translation structure visualizes the decoding of the first tmRNA alanine codon

**David Teran^1^, Ying Zhang^1,2^ and Andrei A. Korostelev^1^***


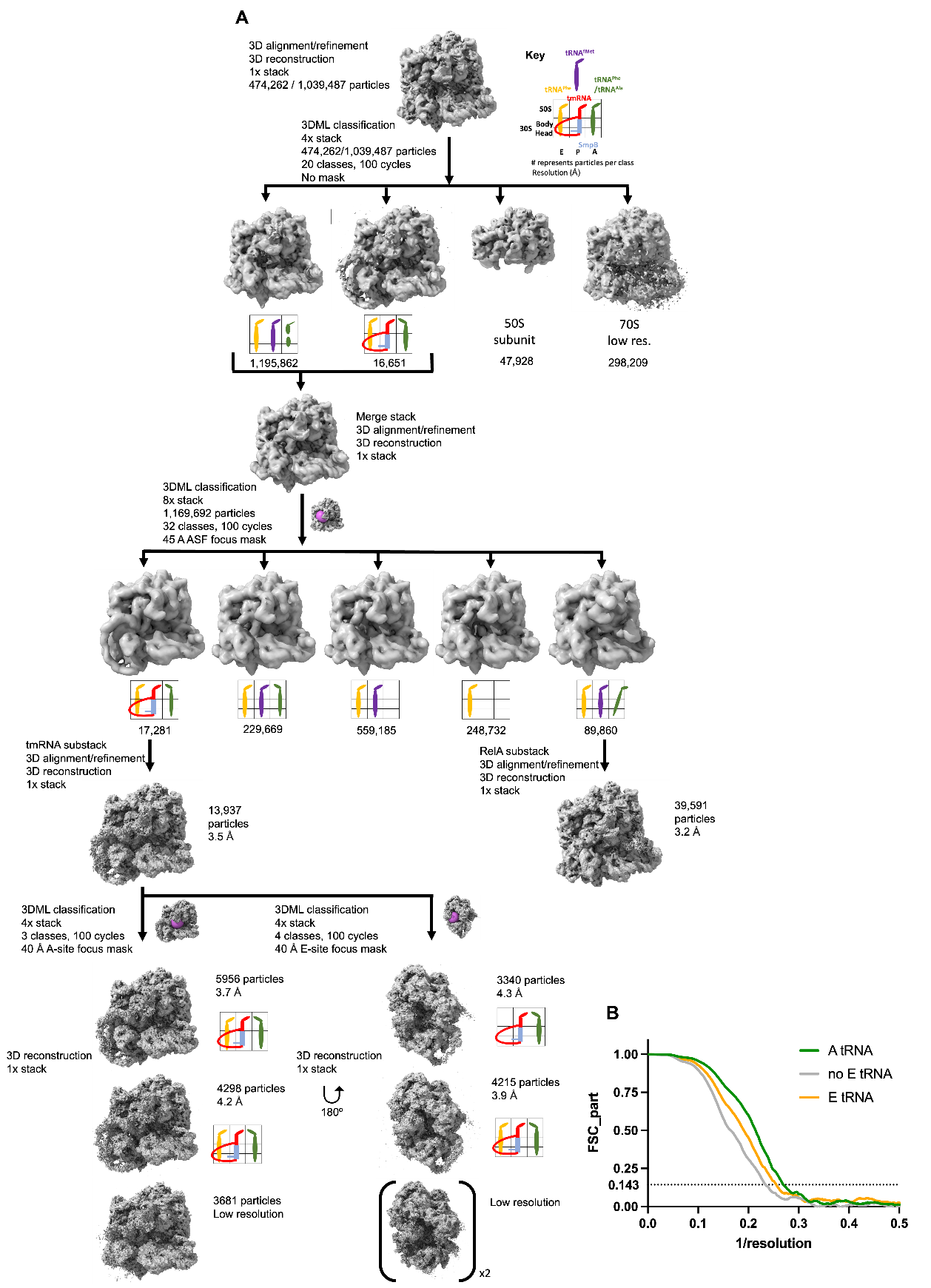


**Figure S1**. Cryo-EM analyses of the *E. coli* 70S datasets. (**A**) Maximum-likelihood classification in Frealign reveals a class with tmRNA bound to the ribosomal P site and tRNA bound to the A site. Additional classification separates ribosome particles with and without E-site tRNA. A magenta sphere illustrates a spherical mask used for classifications. (**B**) Fourier shell correlation curves (FSC) as a function of inverse resolution for the resulting cryo-EM maps.


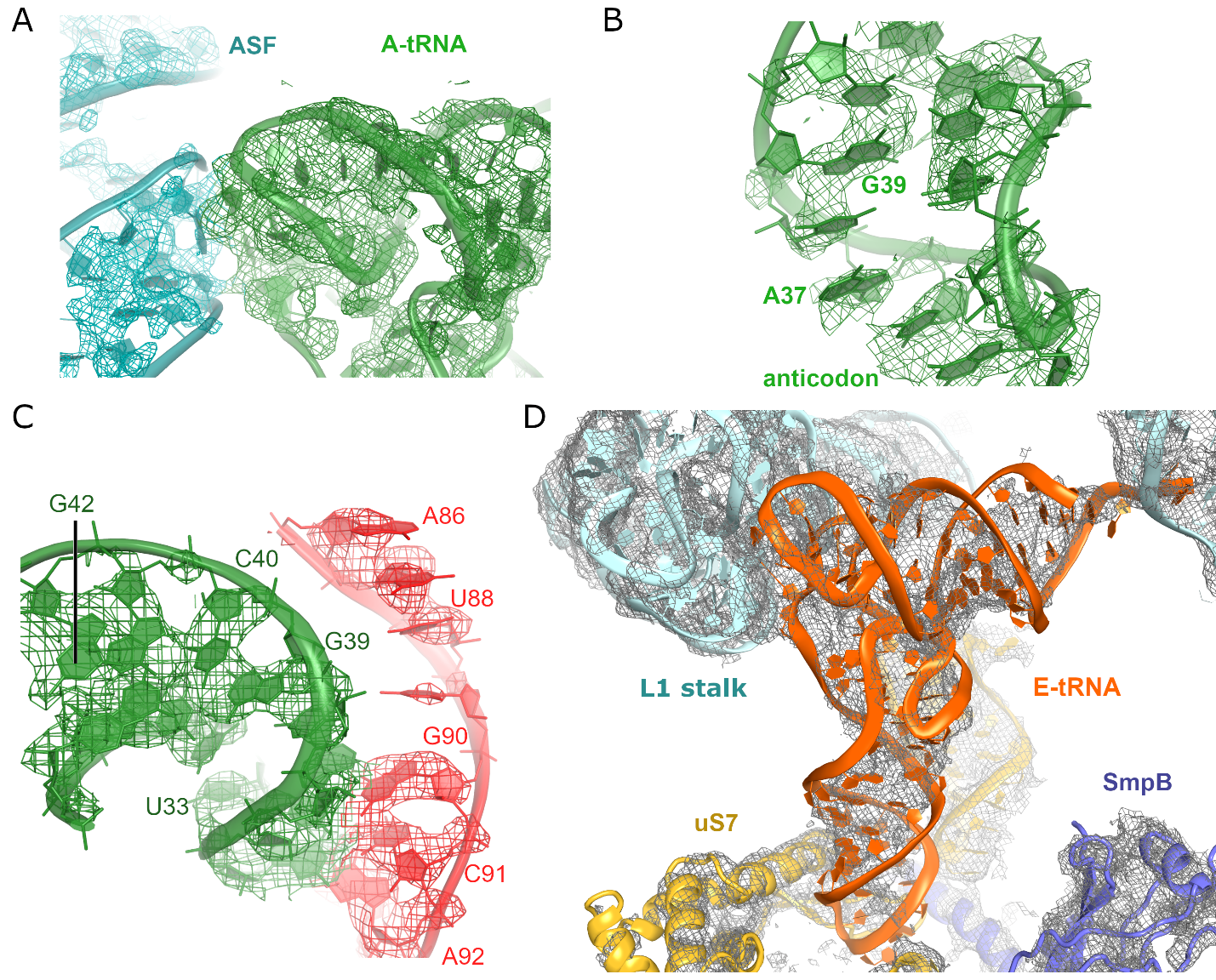


**Figure S2**. Cryo-EM density (mesh) for A-site and E-site tRNA interactions. (**A**) A-tRNA elbow binds next to the A-site finger (ASF) of 23S rRNA. (**B**) Anticodon stem loop of A-site tRNA^Ala^ resolved in the cryo-EM map. (**C**) Nucleotides of the anticodon stem of tRNA^Ala^ and the MLD of tmRNA. (**D**) tRNA binding in the E site near the L1 stalk, uS7 and C-terminal helix of SmpB.

**Supplementary Table S1.** Cryo-EM data collection, structure refinement and stereochemistry statistics

| **Data collection and processing** | A-tRNA | E-tRNA / A-tRNA |
| --- | --- | --- |
| Magnification | 45,000x | 45,000x |
| Voltage (kV) | 200 | 200 |
| Electron exposure (e^-^/Å^2^) | 29.9: 30.4 | 29.9: 30.4 |
| Defocus range (μm) | -0.5-1.5 | -0.5-1.5 |
| Pixel size (Å) | 0.87 | 0.87 |
| Symmetry imposed | C1 | C1 |
| Initial particle images (no.) | 1,513,749 | 1,513,749 |
| Final particles images (no.) | 5956 | 4215 |
| Map resolution (Å)^**^ | 3.7 | 3.9 |
| FSC threshold | 0.143 | 0.143 |
| **Refinement** |  |  |
| Initial model used (PDB code) | 6WDE/6Q98/7ACJ | 6WDE/6Q98/7ACJ |
| Model resolution (Å) | 3.4 | 3.8 |
| Correlation Coefficient (CC_mask)^*^ | 0.761 | 0.741 |
| Map-sharpening *B* factor (Å^2^) | -100 | -50 |
| Model composition^*^ |  |  |
| Non-hydrogen atoms | 155,852 | 157,492 |
| Protein residues | 6218 | 6218 |
| RNA residues | 5001 | 5078 |
| R.m.s deviations^*,^ § |  |  |
| Bond lengths (Å) | 0.0040 | 0.0025 |
| Bond angles (°) | 0.72 | 0.57 |
| Validation |  |  |
| MolProbity score | 2.91 | 2.74 |
| Clashscore | 9.58 | 9.48 |
| Poor rotamers (%) | 13.58 | 9.56 |
| Ramachandran plot |  |  |
| Favored (%) | 90.33 | 91.82 |
| Allowed (%) | 9.67 | 8.18 |
| Disallowed (%) | 0.00 | 0.00 |
| RNA^#^ |  |  |
| Average suiteness | 0.375 | 0.422 |

** from Frealign (FSC_part)

* from Phenix

§ root mean square deviations from ideal values

^#^ RNA backbone suites as defined by Molprobity
